# Shining a Light Inside the “Black Box” of NIH Application Submission and Review

**DOI:** 10.1093/geroni/igab046.2815

**Published:** 2021-12-17

**Authors:** Elia Ortenberg, Shalanda Bynum

**Affiliations:** 1 National Institutes of Health, Bethesda, Maryland, United States; 2 Center for Scientific Review (CSR) National Institutes of Health (NIH), Bethesda, Maryland, United States

## Abstract

What happens to applications after they are submitted to the National Institutes of Health, and how can you better prepare yourself and your application for the process of peer review? The Center for Scientific Review (CSR) works closely with the 24 funding institutes and centers at the National Institutes of Health that provide funding support for projects of high scientific merit and high potential impact. CSR conducts the first level of review for the majority of grant applications submitted to the NIH, which includes 90% of R01s, 85% of Fellowships, and 95% of Small Business Innovation Research (SBIR) applications as well as many other research and training opportunity activities. In this capacity, CSR helps to identify the most meritorious projects, cutting-edge research, and future scientists who will advance the mission of the NIH: to enhance health, lengthen life, and reduce illness and disability. The purpose of this project is to provide an overview of 1) what happens to NIH applications before, during, and after peer review at CSR; 2) a summary of new and current peer review policies and practices that impact investigators and their submitted applications; and 3) strategies for developing a strong NIH grant application. Peer review is the cornerstone of the NIH grant supporting process, and an insider’s view can shine a light inside the “Black Box” of how the most meritorious projects are identified.

